# Supplementary material for: Molecular basis for receptor recognition and broad host tropism for merbecovirus MjHKU4r-CoV-1
Source: EMBO Rep. 2024 Jun 14;25(7):17. doi: 10.1038/s44319-024-00169-8 (PMC11239678; doi:10.1038/s44319-024-00169-8)
Supplement: Supplementary file 7 — Expanded View Figures [file 44319_2024_169_MOESM7_ESM.pdf]

Expanded View Figures

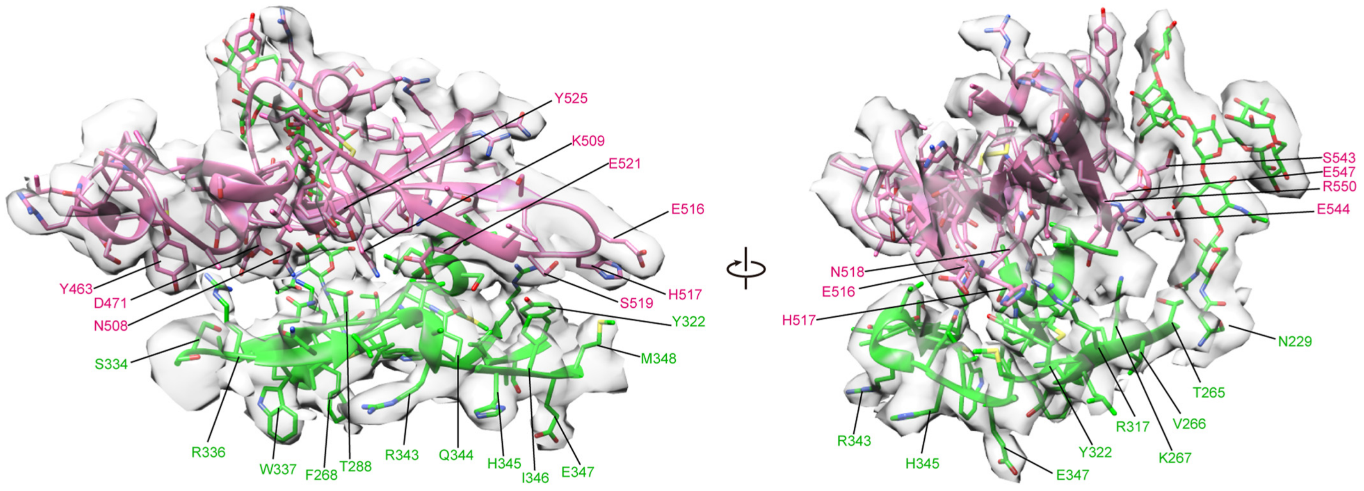

Figure EV1. Local density map of the interface between the MjHKU4r-CoV-1 RBD and hCD26.

The MjHKU4r-CoV-1 RBD and hCD26 are colored in hot pink and green, respectively. The local 2Fo-Fc map contoured at 0.5σ for its binding interface is shown as gray surfaces, and AAs are displayed as sticks.

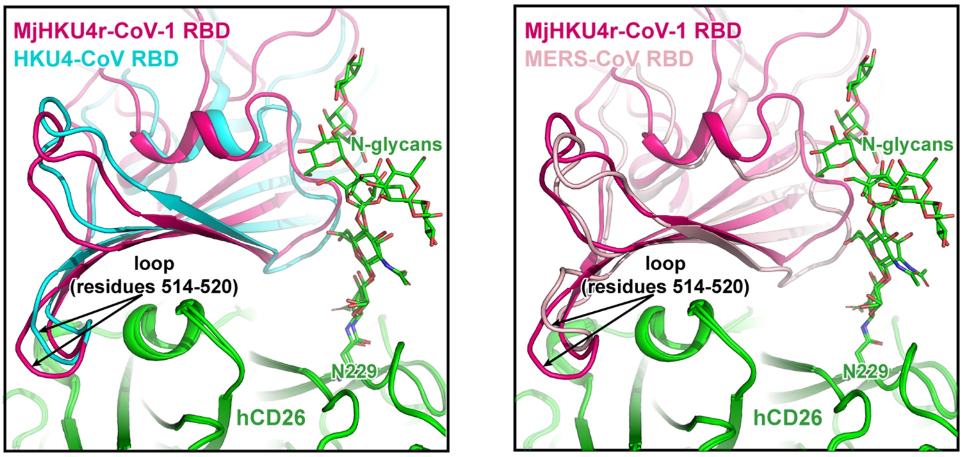

Figure EV2. Structural comparisons among the MjHKU4r-CoV-1 RBD/hCD26, HKU4-CoV RBD/hCD26, and MERS-CoV RBD/hCD26 complexes.

The MjHKU4r-CoV-1, HKU4-CoV, and MERS-CoV RBDs and hCD26 are colored corresponding to Fig. 4.

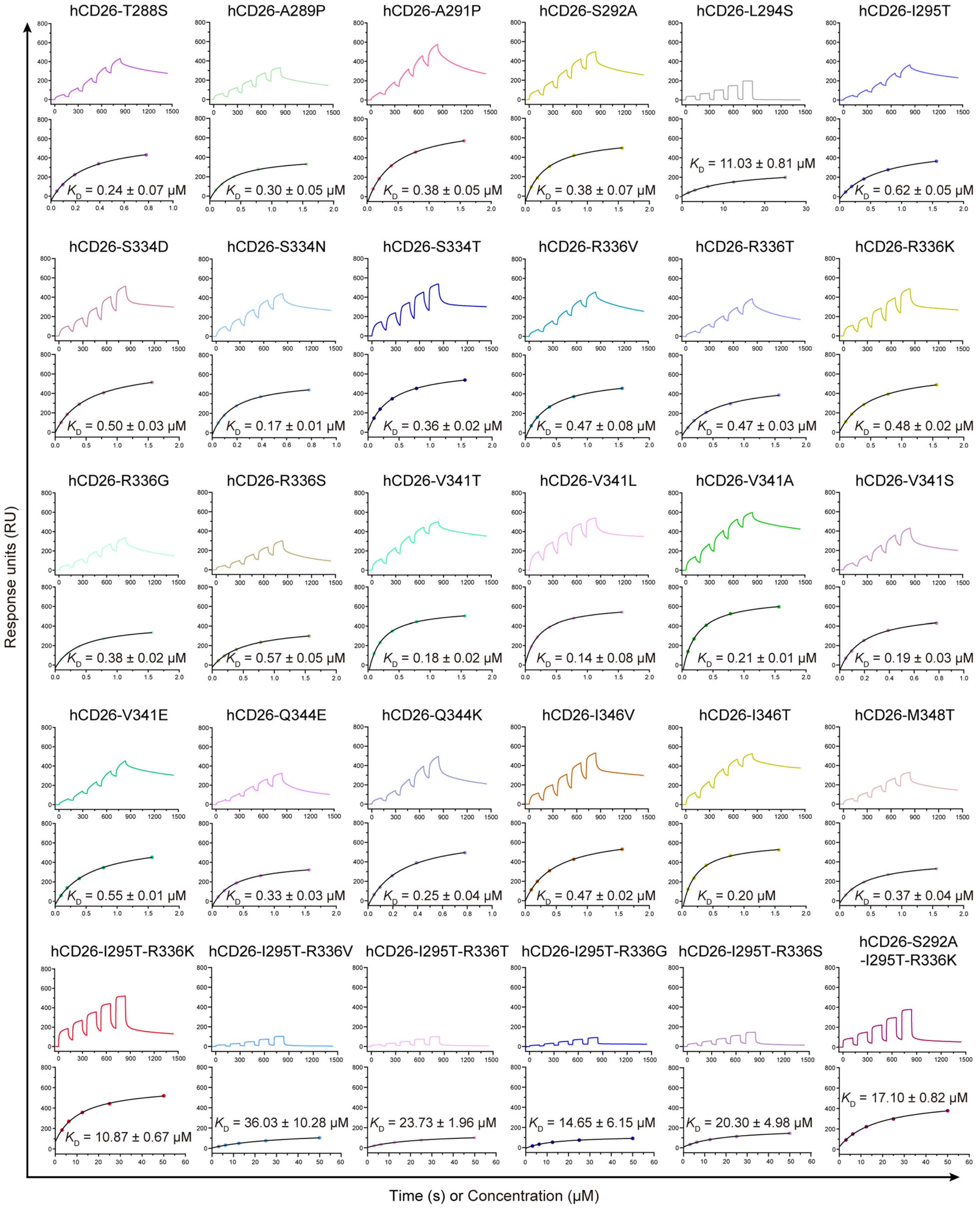
hCD26-T288S
K_D = 0.24 ± 0.07 µM
hCD26-A289P
K_D = 0.30 ± 0.05 µM
hCD26-A291P
K_D = 0.38 ± 0.05 µM
hCD26-S292A
K_D = 0.38 ± 0.07 µM
hCD26-L294S
K_D = 11.03 ± 0.81 µM
hCD26-I295T
K_D = 0.62 ± 0.05 µM
hCD26-S334D
K_D = 0.50 ± 0.03 µM
hCD26-S334N
K_D = 0.17 ± 0.01 µM
hCD26-S334T
K_D = 0.36 ± 0.02 µM
hCD26-R336V
K_D = 0.47 ± 0.08 µM
hCD26-R336T
K_D = 0.47 ± 0.03 µM
hCD26-R336K
K_D = 0.48 ± 0.02 µM
hCD26-R336G
K_D = 0.38 ± 0.02 µM
hCD26-R336S
K_D = 0.57 ± 0.05 µM
hCD26-V341T
K_D = 0.18 ± 0.02 µM
hCD26-V341L
K_D = 0.14 ± 0.08 µM
hCD26-V341A
K_D = 0.21 ± 0.01 µM
hCD26-V341S
K_D = 0.19 ± 0.03 µM
hCD26-V341E
K_D = 0.55 ± 0.01 µM
hCD26-Q344E
K_D = 0.33 ± 0.03 µM
hCD26-Q344K
K_D = 0.25 ± 0.04 µM
hCD26-I346V
K_D = 0.47 ± 0.02 µM
hCD26-I346T
K_D = 0.20 µM
hCD26-M348T
K_D = 0.37 ± 0.04 µM
hCD26-I295T-R336K
K_D = 10.87 ± 0.67 µM
hCD26-I295T-R336V
K_D = 36.03 ± 10.28 µM
hCD26-I295T-R336T
K_D = 23.73 ± 1.96 µM
hCD26-I295T-R336G
K_D = 14.65 ± 6.15 µM
hCD26-I295T-R336S
K_D = 20.30 ± 4.98 µM
hCD26-S292A-I295T-R336K
K_D = 17.10 ± 0.82 µM
Response units (RU)
Time (s) or Concentration (µM)

Figure EV3. Identification of crucial residue determinants for the host range of MjHKU4r-CoV-1.

SPR analysis of binding between the MjHKU4r-CoV-1 RBD and hCD26 mutants: hCD26-T288S, A289P, A291P, S292A, L294S, I295T, S334D/N/T, R336V/T/K/G/S, V341T/L/A/S/E, Q344E/K, I346V/T, M348T, I295T-R336K, I295T-R336V, I295T-R336T, I295T-R336G, I295T-R336S, and S292A-I295T-R336K. Raw curves are shown as the indicated colors. The fit curves are represented by black lines. K_D values are the mean ± SD of three biological replicates. Source data are available online for this figure.

 © The Author(s)

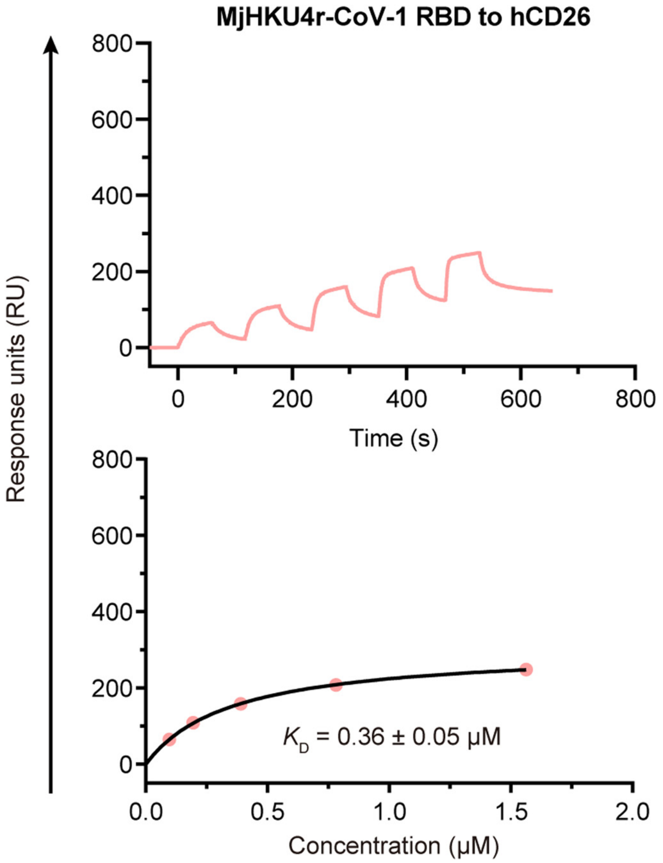

Figure EV4. Binding affinity measurement between the MjHKU4r-CoV-1 RBD and hCD26 using a capture method.

Raw curves are shown in pink, and the fit curves are represented by black lines. K_D values are the mean ± SD of three biological replicates. Source data are available online for this figure.

© The Author(s)
